# Reaching people through medical humanities: An initiative

**DOI:** 10.3352/jeehp.2011.8.5

**Published:** 2011-05-20

**Authors:** Richa Gupta, Satendra Singh, Mrinalini Kotru

**Affiliations:** 1 Department of Pathology, Chacha Nehru Bal Chikitsalaya, Delhi, India.; 2 Department of Physiology, University College of Medical Sciences, Delhi, India.; 3 Medical Humanities Group, University College of Medical Sciences, Delhi, India.; 4 Department of Pathology, University College of Medical Sciences, Delhi, India.

Medical humanities (MH) has been defined as "an interdisciplinary and increasingly international endeavour that draws on the creative and intellectual strengths of diverse disciplines including literature, art, creative writing, drama, film, music, philosophy, ethical decision making, anthropology, and history in pursuit of medical educational goals" [1]. As newer treatment modalities and yet new ailments surface, the need for a more holistic approach towards healing and cure has been emphasised. Besides, it has been increasingly felt that the present doctors lack empathy and compassion for their patient. This may be partly attributed to the growing socio economic burden but is also due to the fact that the present medical curriculum does not include any such subject which relates to the conduct of the health care providers. Engagement with humanities offers many benefits, including fostering clinicians' abilities to communicate with patients, to penetrate more deeply into the patient's wider narrative, and to seek more diverse ways of promoting well being and reducing the impact of illness or disability [2]. Oyebode [3] has illustrated different ways in which humanities can enrich medical practice. To put it simply, engagement in such activities may lead to overall behavioural change of medical professionals thus improving their approach towards the patient.

MH programs are common in the United States and most of the European countries. In certain schools MH is a voluntary module while in others it is a part of the curriculum. Many medical schools offer a number of elective MH courses and students can select a particular one according to their interest and aptitude. However, the idea of MH is still relatively new in Asia. Two teaching modules of MH were conducted in Nepal and the authors found a positive approach of students towards the modules [4,5]. While the problem of lack of communication between the doctors and the patients has long been recognised in India as well, not much has been done to rectify it. MH is largely unknown in most of the medical schools and institutions in India. Also, we are not aware of any teaching modules or programmes on MH in the country.

We have formed a Medical Humanities Group as a part of Medical Education Unit of University College of Medical Sciences with the aim to develop this discipline and its practice amongst health care providers. The target population includes doctors, medical students, paramedical staff, nurses and technicians. We expect that this would in turn perk up their communication skills and lead to a better understanding of disease suffering. In an effort to integrate it into the current social and educational system, we have begun a series of lectures under the name "Confluence." Five events under this series have been conducted till date. The events are conducted during lunch hour so that maximum number of people may be involved without disrupting daily work. These events include four interactive lectures and one street play by the medical students.

The lectures were delivered by prominent personalities from different educational, cultural and social fields who shared their vivid experiences. All the events were highly appreciated by a large, convivial audience. A brief description about the five events is given in the following paragraphs. The inaugural lecture "The Doctor is in" was conducted in January 2010. It was delivered by Professor Ramesh Bijlani, who was formerly Head of the Department of Physiology at All India Institute of Medical Sciences (AIIMS) and is currently involved in teaching yoga at Sri Aurbindo Ashram. Professor Bijlani, who was instrumental in developing Physical Fitness department at AIIMS, discussed the importance of holistic approach to cure ailments, especially chronic diseases. The lecture was accompanied by an art exhibition by Dr. Apurba Rajbongshi, Senior Resident, Pathology, University College of Medical Sciences and Guru Teg Bahadur Hospital. Both the lecture and exhibition were widely appreciated by the audience. The overwhelming response to the inaugural event gave us a lot of positive feedback and encouraged us to peruse the goal of spreading awareness about MH with more vigour and enthusiasm. The second lecture in the series was taken by Mr. Sanal Edmarku in February 2010. He is the president of Indian Rationalist Association and is considered to be the most learned and outspoken speaker on "Rationalism" in India. In his lecture "Faith Under the Scalpel," he tackled the deep-rooted Indian belief in re-incarnation and astrology, and gave rational and scientific explanations for a wide range of "miracles" and alleged power demonstrations of supernatural forces, setting to rest all "mass hysteria" attached with them. The third event in the series was a Street Play by medical students of University College of Medical sciences. The play was called "AIDS - Academic Induced Degenerative Syndrome," and was an eye opener about the increasing burden of studies in medical schools. The play targeted the inadequacies of current medical curriculum which may be the reason for growing personality disorders amongst medical students. This was followed a month later by Professor C. J. Daswani, currently Executive Director, Remedia Trust, taking a lecture on "Communication with the Grassroots." Professor Daswani emphasised the importance of communication in the doctor-patient relationship. He gave several real life examples illustrating how better communication can make a striking differences in the treatment outcomes for the patient. He also expressed grief about the changing face of today's doctors and how they were becoming insensitive to patient complaints relying more on costly investigations instead.

In the most recent event in this series, we organised a "A day for Tibet" on India's Hindi Diwas. This included an interactive session by renowned journalist Mr. Vijay Kranti, a short film about the struggle of Tibetans for freedom and a photo exhibition. Mr. Vijay Kranti, who has more than three decades of experience on this issue, told about the 50 year old Tibetan struggle and how this was relevant to Indian Security. He also talked about the commitments of his holiness Dalai Lama and his persistent efforts towards humanities. To judge the impact of these lectures feedback forms were distributed during all the events as well as a register for remarks was kept at the entrance. The feedback form included carefully framed questions about the liking of the event by the audience, their understanding of humanities and their inputs about how we could improve these lectures. Also, each time certain questions were asked to assess the approach of the audience towards the patients and consequently their behaviour in different situations.

So far our journey of MH and confluence has been smooth, with minor obstacles. The response of the medical fraternity has been positive depicted by the growing number of audience each time. Our innovation is the first of its kind in the country. The Group has been included in the Medical Humanities community in the New York Database [6]. Our final aim is to provide MH with its due importance, setting up of fellowships, degree and diploma courses in MH as in the West, so that the doctors can have a more humanitarian approach towards the patient.
